# Saikosaponin D Inhibits the Proliferation and Promotes the Apoptosis of Rat Hepatic Stellate Cells by Inducing Autophagosome Formation

**DOI:** 10.1155/2021/5451758

**Published:** 2021-08-18

**Authors:** Hong Jiang, Jia Liu, Kun Zhang, Qingxin Zeng

**Affiliations:** ^1^Department of Pathophysiology, Baotou Medical College, Inner Mongolia University of Science and Technology, Baotou, China; ^2^Department of Hepatopancreatobiliary Surgery, Cancer Hospital of China Medical University, Liaoning Cancer Hospital & Institute, Shenyang, China; ^3^Department of Neurology, Baogang Hospital of Inner Mongolia, Baotou, China

## Abstract

**Objective:**

This study aimed to investigate the effects of saikosaponin D (SSd) on the proliferation and apoptosis of the HSC-T6 hepatic stellate cell line and determine the key pathway that mediates SSd's function.

**Methods:**

Cell viability was detected using the CCK-8 kit. The EdU kit and flow cytometry were used to examine cell proliferation. The Annexin V-FITC/PI double staining kit and flow cytometry were used to examine cell apoptosis. Western blot analysis was performed to analyze the expression levels of LC3, Ki67, cleaved caspase 3, Bax, and Bcl2. Autophagosome formation was detected by LC3-GFP adenovirus transfection.

**Results:**

SSd inhibits the proliferation and promotes the apoptosis of acetaldehyde-activated HSC-T6 cells. SSd treatment increased the expression of cleaved caspase 3 and Bax but reduced that of Ki67 and Bcl2. The same concentration of SSd barely influenced the growth of normal rat liver BRL-3A cells. SSd upregulated LC3-II expression and induced autophagosome formation. Autophagy agonist rapamycin had the same effect as SSd and autophagy inhibitor 3-methyladenine could neutralize the effect of SSd in acetaldehyde-activated HSC-T6 cells.

**Conclusions:**

SSd could inhibit the proliferation and promote the apoptosis of HSC-T6 cells by inducing autophagosome formation.

## 1. Introduction

Alcoholic liver disease (ALD) is a general designation for various hepatic injuries caused by long-term heavy alcohol consumption, and it has become a common disease worldwide [1, 2]. The pathologic stages of ALD include steatosis (alcoholic fatty liver), steatohepatitis (alcoholic hepatitis), and liver fibrosis/cirrhosis [3]. Steatosis and steatohepatitis are the early stages of ALD and are considered the precursor lesions of liver fibrosis/cirrhosis [4, 5]. Currently, ALD treatment potentially aims to delay and reverse alcoholic liver fibrosis [6].

Hepatic stellate cells (HSCs) play a key role in the development of alcoholic liver fibrosis because activated HSCs are the primary producers of extracellular matrix (ECM) in the liver [7, 8]. Acetaldehyde, the first metabolite of ethanol oxidation, can stimulate HSCs to proliferate and produce ECM proteins. Therefore, inhibition of HSC proliferation and ECM synthesis is a feasible method for delaying or reversing alcoholic liver fibrosis [9].

*Bupleurum falcatum* is a frequently-used Chinese herb that has anti-inflammatory, anti-infection, and liver-protection effects [10–12]. In our previous study, a complex Chinese herbal prescription containing *B. falcatum* was shown to significantly inhibit CCl_4_-induced liver fibrosis in rats [13]. Saikosaponin D (SSd) is an important active component isolated from *B. falcatum*. Previous studies have demonstrated that SSd inhibits the proliferation of activated T lymphocytes and cancer cells [14–16]. Recently, it has been reported to promote the apoptosis of the HSC-T6 and LX-2 HSC cell lines [17]. However, the molecular mechanism behind SSd's effect on HSCs is not entirely understood.

Autophagy is an evolutionarily conserved lysosomal degradation process essential for maintaining homeostasis in eukaryotic cells [18]. It is involved in various cellular processes such as cell survival, development, proliferation, apoptosis, and differentiation [19]. Several compounds have been reported to modulate autophagy, and some have shown potential therapeutic effects for various diseases [20–22]. Recently, SSd has been reported to induce autophagosome formation [22–24].

This study aimed to verify if SSd could induce autophagosome formation in HSCs and if autophagosome formation is the key pathway involved in proliferation inhibition and apoptosis promotion in HSCs.

## 2. Materials and Methods

### 2.1. Reagents

SSd (HY-N0250), rapamycin (HY-10219), and 3-methyladenine (3MA; HY-19312) were purchased from MedChemExpress. LC3B (18725-1-AP), Bax (50599-2-Ig), Bcl2 (26593-1-AP), and beta-actin (20536-1-AP) antibodies were purchased from ProteinTech. Ki67 (ab16667) antibodies were purchased from Abcam. Cleaved caspase 3 (#9661) antibodies were purchased from Cell Signaling Technology.

### 2.2. Cell Cultures and Treatments

HSC-T6 cells, an immortalized rat HSC line, were brought from Shanghai YSRIBIO. BRL-3A cells, an immortalized rat liver cell line, were obtained from the China National Collection of Authenticated Cell Cultures. They were cultured in a 5% CO_2_-humidified incubator at 37°C in high-glucose Dulbecco's modified eagle's medium (Gibco, USA), supplemented with 10% fetal bovine serum (Gibco), 100 U/mL penicillin (Solarbio, China), and 100 U/mL streptomycin (Solarbio).

### 2.3. Cell Viability Measurement

Cell viability was determined using CCK-8 kits (Beyotime, China). In brief, HSC-T6 cells were seeded in 96-well plates (Corning, USA) at a concentration of 5 × 10^3^ cells per well, allowed to adhere overnight and subsequently treated with various concentrations of acetaldehyde or SSd for 24 or 48 h as previously described [25]. After treatment with acetaldehyde, 10 *μ*l CCK-8 was added to each well and the cells were incubated for 1 h. The absorbance was measured at 450 nm using a microplate reader.

### 2.4. 5-Ethynyl-2′-deoxyuridine Assays

To determine cell proliferation, 2 × 10^5^ cells/well were plated on a 6-well plate overnight and then treated with various interventional drugs for 48 h. After treatment, the 5-ethynyl-2′-deoxyuridine (EdU) reagent (C0075S, Beyotime) was added to each well and the cells were incubated for 3 h. The cells were then digested into a single-cell suspension and fixed in 4% paraformaldehyde for 15 min. Thereafter, at least 1 × 10^4^ cells were detected using a flow cytometer (BD Biosciences, USA). Finally, the FlowJo software (Tree Star Inc., USA) was used to analyze the results.

### 2.5. Apoptosis Assay

Cell apoptosis was assessed using the Annexin V-fluorescein isothiocyanate (FITC) apoptosis detection kit (KeyGen Biotech, China). A total of 2 × 105 cells/well were plated on a 6-well plate, incubated overnight, and then treated with various interventional drugs for 48 h. Cells were then digested using 0.25% trypsin without EDTA into a single-cell suspension. According to the manufacturer's protocols, Annexin V-FITC and propidium iodide (PI) were used for cell staining. Then, at least 2 × 10^4^ double-stained cells were measured using a flow cytometer (BD Biosciences). Finally, the FlowJo software was used to analyze the results.

### 2.6. GFP-LC3 Adenovirus Transfection

As previously described [26], cells in the confocal dishes were transfected with GFP-LC3 adenovirus (Hanbio, China) at a multiplicity of infection of 80. After 48 h of transfection, the cells were treated with various interventional drugs (DMSO, 10 *μ*mol/L SSd, and 10 *μ*mol/L SSd combined with 5 mmol/L 3MA) for 6 h. Then, GFP-LC3 dots in the cells were observed using a confocal microscope.

### 2.7. Western Blot Analysis

Western blot analysis was performed based on standard protocols. In brief, the total protein from cells was extracted using the RIPA buffer (Beyotime) and protein concentrations were determined using the BCA kit (Beyotime). Proteins were then separated by electrophoresis and electrotransferred onto a PVDF membrane. Afterward, the membranes were blocked with 5% nonfat milk (Boster Biological Technology, China) for 30 min and incubated with LC3B (1 : 1000 dilution), Ki67 (1 : 1000), cleaved caspase 3 (1 : 1000), Bcl2 (1 : 1000), Bax antibody (1 : 1000), and beta-actin (1 : 4000) antibodies overnight at 4°C, followed by the incubation with horseradish peroxidase-conjugated secondary antibodies for 1.5 h at 20°C. Proteins were visualized using the ECL reagent (Beyotime). The results were obtained using the Bio-Rad Gel Doc XR+ System.

### 2.8. Statistical Analysis

Data are presented as mean ± standard deviation. The differences among the means and the effects of treatments were analyzed using student's unpaired *t*-test with two-tailed *p* values and one-way ANOVA followed by Tukey's multiple comparisons test, using GraphPad Prism 7 (GraphPad Software, USA). Statistical significance was set at *p* < 0.05. All experiments were performed at least thrice.

## 3. Results

### 3.1. Effects of SSd on HSC-T6 Cell Proliferation

**Figure 1:**
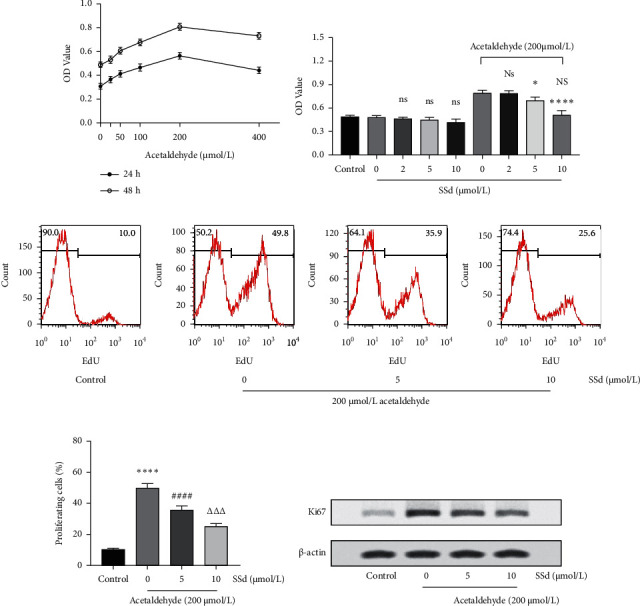
Effects of SSd on HSC-T6 cell proliferation. (a) HSC-T6 cell viability was detected after 25, 50, 100, 200, or 400 *μ*mol/L acetaldehyde treatment for 24 or 48 h using the CCK-8 kit. (b) Cell viability of nonactivated and acetaldehyde-activated HSC-T6 cells was detected after 2, 5, or 10 *μ*mol/L SSd treatment for 24 h using the CCK-8 kit. The ns means no significant difference versus 0 *μ*mol/L SSd-treated nonactivated HSC-T6 cells. The Ns means no significant difference versus 0 *μ*mol/L SSd-treated acetaldehyde-activated HSC-T6 cells. ^*∗*^*p* < 0.05, ^*∗∗∗∗*^*p* < 0.0001 versus 0 *μ*mol/L SSd-treated acetaldehyde-activated HSC-T6 cells. The NS means no significant difference versus control group cells. (c) Acetaldehyde-activated HSC-T6 cell proliferation was detected under SSd treatment for 48 h using the EdU kit and flow cytometry. (d) The percentage of proliferating cells based on EdU flow cytometry detection. ^*∗∗∗∗*^*p* < 0.0001 versus control group (nonactivated HSC-T6 cells); ^####^*p* < 0.0001 versus 0 *μ*mol/L SSd treatment group; ^ΔΔΔ^*p* < 0.001 versus 5 *μ*mol/L SSd treatment group. (b, d) Data are presented as mean ± SD (*n* = 3). One-way ANOVA and then Tukey's multiple comparisons test were used for statistical analysis. (e) The expression level of Ki67 in acetaldehyde-activated HSC-T6 cells was detected by western blot analysis after 48 h of SSd treatment.

CCK-8 was used to detect the effects of acetaldehyde and SSd on HSC-T6 cell viability. The results (Figure 1(a)) showed that 200 *μ*mol/L acetaldehyde had the strongest promotion effect on HSC-T6 cell proliferation. Thus, 200 *μ*mol/L acetaldehyde-activated HSC-T6 cells were used for follow-up studies. SSd barely influenced the cell viability of nonactivated HSC-T6 cells, whereas it inhibited the cell viability of activated HSC-T6 cells (Figure 1(b)). Furthermore, 10 *μ*mol/L SSd could almost completely inhibit the proproliferation effect of 200 *μ*mol/L acetaldehyde. The EdU proliferation test showed that approximately 50% of acetaldehyde-activated HSC-T6 cells were in the DNA-replication state, and SSd could significantly inhibit the proliferation of activated HSC-T6 cells (Figures 1(c) and 1(d)). However, SSd barely influenced the proliferation of nonactivated HSC-T6 cells in the EdU test (Supplementary Figure S1). Western blot analysis showed that acetaldehyde significantly increased Ki67 expression in HSC-T6 cells, whereas SSd significantly inhibited Ki67 expression in these cells (Figure 1(e)).

### 3.2. Effects of SSd on HSC-T6 Cell Apoptosis

**Figure 2:**
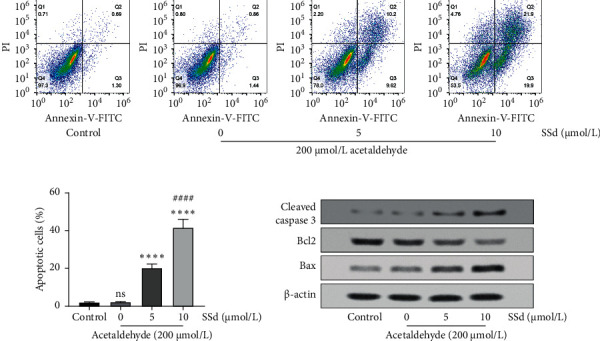
Effects of SSd on HSC-T6 cell apoptosis. (a) Acetaldehyde-activated HSC-T6 cell apoptosis was detected after SSd treatment for 48 h using the Annexin V-FITC/PI double-stained kit and flow cytometry. (b) The percentage of apoptotic cells based on Annexin V-FITC/PI double-stained flow cytometry results. The ns means no significant difference versus control group (nonactivated HSC-T6 cells). ^*∗∗∗∗*^*p* < 0.0001 versus 0 *μ*mol/L SSd treatment group; ^####^*p* < 0.0001 versus 5 *μ*mol/L SSd treatment group. Data are presented as mean ± SD (*n* = 3). One-way ANOVA and then Tukey's multiple comparisons test were used for statistical analysis. (c) The expression levels of cleaved caspase 3, Bcl2, and Bax in acetaldehyde-activated HSC-T6 cells were detected by western blot analysis after 48 h of SSd treatment.

The results of Annexin V-FITC/PI double staining revealed that SSd significantly promoted acetaldehyde-activated HSC-T6 cell apoptosis (Figures 2(a) and 2(b)). However, it barely influenced the apoptosis of nonactivated HSC-T6 cells (Supplementary Figure S2). Western blot analysis showed that SSd promoted acetaldehyde-activated HSC-T6 cell apoptosis by increasing the expression of cleaved caspase 3 and Bax and reducing the expression of Bcl2 (Figure 2(c)).

### 3.3. Effects of SSd on Rat Liver BRL-3A Cells

**Figure 3:**
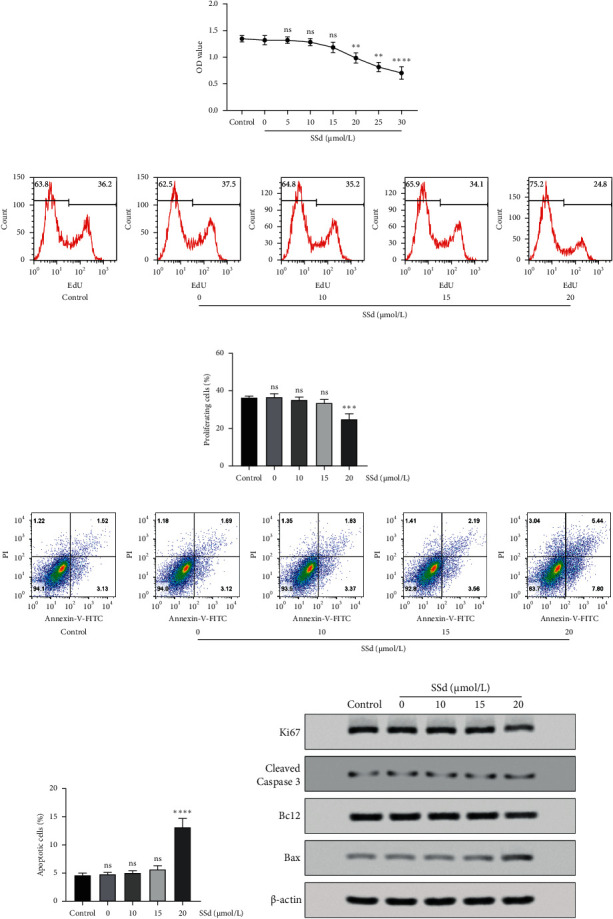
Effects of SSd on rat liver BRL-3A cells. (a) BRL-3A cell viability was detected after 5, 10, 15, 20, 25, and 30 *μ*mol/L SSd treatment for 48 h using the CCK-8 kit. The ns means no significant difference versus 0 *μ*mol/L SSd-treated BRL-3A cells. ^*∗∗*^*p* < 0.01, ^*∗∗∗∗*^*p* < 0.0001 versus 0 *μ*mol/L SSd-treated BRL-3A cells. (b) BRL-3A cell proliferation was detected after 10, 15, and 20 *μ*mol/L SSd treatment for 48 h using the EdU kit and flow cytometry. (c) The percentage of proliferating cells based on EdU flow cytometry results. The ns means no significant difference versus control group. ^*∗∗∗∗*^*p* < 0.001 versus control group. (d) BRL-3A cell apoptosis was detected after 10, 15, and 20 *μ*mol/L SSd treatment for 48 h using the Annexin V-FITC PI double-stained kit and flow cytometry. (e) The percentage of apoptotic cells based on Annexin V-FITC PI double-stained flow cytometry results. The ns means no significant difference versus control group. ^*∗∗∗∗*^*p* < 0.0001 versus control group. (c, e) Data are presented as mean ± SD (*n* = 3). One-way ANOVA and then Tukey's multiple comparisons test were used for statistical analysis. (f) Ki67, cleaved caspase 3, Bcl2, and Bax expression levels of BRL-3A cells were detected by western blot analysis after 48 h of SSd treatment.

Analysis using the CCK-8 kit revealed that SSd started to influence the cell viability of BRL-3A cells when its concentration reached 20 *μ*mol/L (Figure 3(a)). The EdU proliferation test showed that it barely inhibited BRL-3A proliferation until the concentration reached 20 *μ*mol/L (Figures 3(b) and 3(c)). Flow cytometry apoptosis test confirmed that 10 *μ*mol/L and 15 *μ*mol/L SSd barely promoted BRL-3A apoptosis (Figures 3(d) and 3(e)). Furthermore, western blot analysis showed that the effective concentration of SSd was 20 *μ*mol/L. When the concentration reached 20 *μ*mol/L, SSd inhibited BRL-3A proliferation by downregulating Ki67 expression and promoted BRL-3A apoptosis by increasing the expression of cleaved caspase 3 and Bax and reducing the expression of Bcl2 (Figure 3(f)). BRL-3A cells were more resistant to SSd than HSC-T6 cells. Furthermore, although 10 *μ*mol/L SSd significantly influenced the proliferation and apoptosis of activated HSC-T6 cells, it barely influenced those of BRL-3A cells. Therefore, we selected 10 *μ*mol/L SSd for follow-up studies.

### 3.4. SSd Inhibits the Proliferation and Promotes the Apoptosis of HSC-T6 Cells via Autophagosome Formation

**Figure 4:**
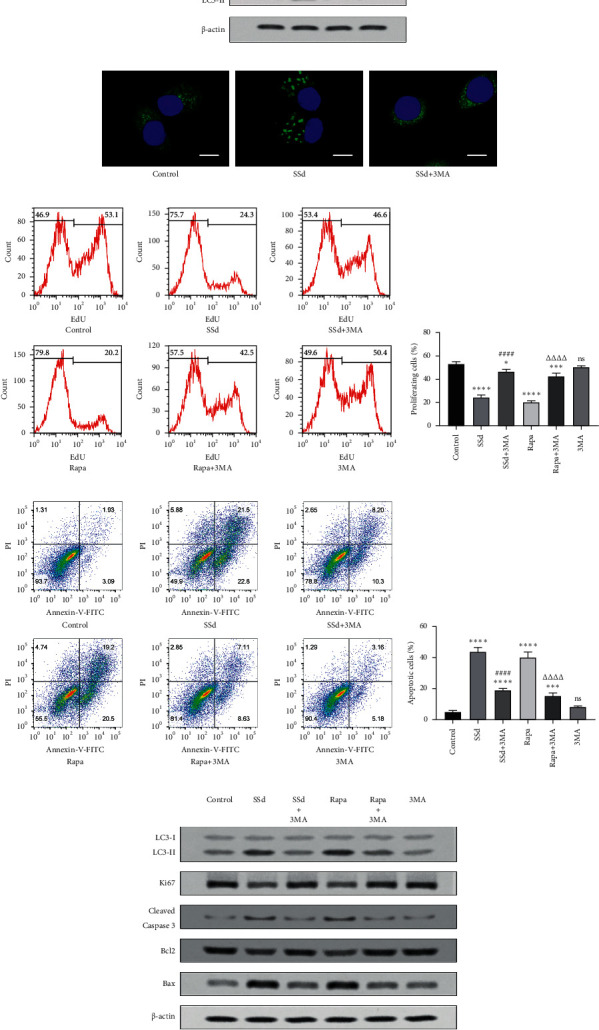
SSd inhibits the proliferation and promotes the apoptosis of HSC-T6 cells via autophagosome formation. (a) LC3B expression level in acetaldehyde-activated HSC-T6 cells was detected by western blot analysis after 6 h of SSd treatment. (b) HSC-T6 cells were transfected using GFP-LC3 adenovirus. GFP-LC3 puncta formation was mediated by SSd in acetaldehyde-activated HSC-T6 cells. Bar: 10 *μ*m. (c) Acetaldehyde-activated HSC-T6 cells were incubated with 10 *μ*mol/L SSd, 10 *μ*mol/L rapamycin, and 5 mmol/L 3MA alone or in combination for 48 h. Cell proliferation was detected using the EdU kit and flow cytometry. (d) The percentage of proliferating cells based on EdU flow cytometry results. ^*∗*^*p* < 0.05, ^*∗∗∗*^*p* < 0.001, ^*∗∗∗∗*^*p* < 0.0001 versus control group; ^####^*p* < 0.0001 versus SSd treatment group; ^ΔΔΔΔ^*p* < 0.0001 versus rapamycin treatment group. The ns means no significant difference versus control group. (e) Acetaldehyde-activated HSC-T6 cells were incubated with 10 *μ*mol/L SSd, 10 *μ*mol/L rapamycin, and 5 mmol/L 3MA alone or in combination for 48 h. Cell apoptosis was detected using the Annexin V-FITC/PI double-stained kit and flow cytometry. (f) The percentage of apoptotic cells based on Annexin V-FITC/PI double-stained flow cytometry results. ^*∗∗∗*^*p* < 0.001, ^*∗∗∗∗*^*p* < 0.0001 versus control group; ^####^*p* < 0.0001 versus SSd treatment group; ^ΔΔΔΔ^*p* < 0.0001 versus rapamycin treatment group. The ns means no significant difference versus control group. (d, f) Data are presented as mean ± SD (*n* = 3). One-way ANOVA and then Tukey's multiple comparisons test were used for statistical analysis. (g) The expression levels of LC3B, Ki67, cleaved caspase 3, Bcl2, and Bax were detected by western blot analysis.

On the basis of reports regarding the effects of SSd on autophagy, we speculate that autophagy is an important target pathway for SSd to influence the proliferation and apoptosis of activated HSC-T6 cells. Western blot analysis revealed that the expression of LC3-II increased with the increase in SSd concentration (Figure 4(a)). We further used HSC-T6 cells with GFP-LC3 transfection to determine the status of the autophagosomes under SSd treatment. We found that SSd induced autophagosome formation, whereas the autophagy inhibitor 3MA could neutralize this effect of SSd (Figure 4(b)). EdU proliferation detection showed that the autophagy inhibitor 3MA could significantly offset the antiproliferation effect of SSd and agonistic effect of rapamycin (Figures 4(c) and 4(d)). Annexin V-FITC/PI double-stained apoptosis detection revealed that 3MA significantly offsets the proapoptotic effects of SSd and rapamycin (Figures 4(e) and 4(f)). Western blot analysis showed that SSd and rapamycin inhibited the expression of Ki67 and Bcl2 and promoted the expression of cleaved caspase 3 and Bax. The autophagy inhibitor 3MA can significantly offset the effects of SSd and rapamycin on the expression of proliferation and apoptosis-related proteins (Figure 4(g)).

## 4. Discussion

Liver fibrosis is primarily caused by the activation of HSCs and high levels of *α*-smooth muscle actin and collagen types I and III [17, 27, 28]. Previous studies have shown that SSd could defend CCl_4−_and dimethylnitrosamine-induced liver injury in rats [29–31] and thus opened the possibility of using SSd in the treatment of liver fibrosis without serious adverse reactions. SSd alleviates liver fibrosis by inhibiting the proliferation and promoting the apoptosis of HSCs [17, 32]. However, the key signaling pathways of SSd that link HSC proliferation and apoptosis remain unclear. Our study verified that SSd inhibited the proliferation and induced the apoptosis of acetaldehyde-activated HSC-T6 cells. More importantly, the pathway involved in the induction of autophagosome formation was the main signaling pathway through which SSd inhibited the proliferation and promoted the apoptosis of HSC-T6 cells.

In Chinese pharmacopoeia, *B. falcatum* is documented as a mixture of dried roots of *B. chinense* DC. and *B. scorzonerifolium* Willd. [33]. *B. falcatum* is a frequently-used Chinese herb that has anti-inflammatory, anti-infection, and liver-protection effects [10–12]. In our previous study, a complex Chinese herbal prescription containing *B. falcatum* could significantly inhibit CCl_4_-induced liver fibrosis in rats [13]. SSd is an active and structurally steroid-like saponin found in *B. falcatum*, and it has been reported to inhibit thioacetamide-induced liver fibrosis and inflammation in mice [33]. However, the mechanism of SSd blocking liver fibrosis remains to be fully elucidated.

SSd has a broad-spectrum of proapoptotic and antiproliferation effects and it has been reported to induce cell apoptosis in a variety of cells, such as lung cancer [34], breast cancer [35], pancreatic cancer [36], multiple myeloma [37], myeloid leukemia [37], glioblastoma [38], liver cancer [39], and prostate cancer [40] cells through various molecular mechanisms. SSd has also been reported to inhibit the proliferation of HSCs via the ERK1/2 pathway [32] and promote their apoptosis via caspase-3-dependent, caspase-3-independent, and mitochondrial pathways [17]. However, the upstream molecular mechanism of inhibiting HSC proliferation and promoting HSC apoptosis remains unclear.

A basal level of autophagy ensures cell survival in the presence of damage or stressful factors. However, the excess accumulation of autophagosomes may become an alternative pathway of cell death. The activation of autophagy occurs earlier than cell apoptosis, placing autophagy upstream of apoptosis. Autophagic activation beyond a certain threshold may result in direct autophagic cell death or cell apoptosis via common regulators such as Bcl2 family proteins [41–43]. SSd has a strong autophagy-regulating ability. Recent studies have demonstrated that it induces the formation of autophagosomes in HeLa [22], liver cancer [44], osteosarcoma [23], renal cystic [24], and breast cancer [45] cells. However, Li et al. found that although SSd induces the formation of autophagosomes, it inhibits the fusion of autophagosomes and lysosomes, causing the excess accumulation of autophagosomes [22]. In this study, SSd activated the excess autophagy of HSC-T6 cells, which resulted in inhibiting their proliferation and promoting apoptosis.

In conclusion, SSd can inhibit the proliferation and promote the apoptosis of HSC-T6 cells by inducing autophagosome formation.

## Data Availability

The experimental data used to support the findings of this study are available from the corresponding author upon request.

## Supplementary Materials

Supplementary Materials Figure S1: effects of SSd on nonactivated HSC-T6 cell proliferation. (A) Nonactivated HSC-T6 cell proliferation was detected under SSd treatment for 48 h using EdU kit and flow cytometry. (B) Percentage of proliferating cells based on EdU flow cytometry detection. The ns means no significant difference versus the control group. Data are displayed as mean ± SD, *n* = 3. One-way ANOVA followed by Tukey's multiple comparisons test was used in each statistical analysis. Figure S2: effects of SSd on nonactivated HSC-T6 cell apoptosis. (A) Nonactivated HSC-T6 cell apoptosis was detected under SSd treatment for 48 h using Annexin V-FITC PI double-stained kit and flow cytometry. (B) Percentage of apoptotic cells based on Annexin V-FITC PI double-stained flow cytometry detection. The ns means no significant difference versus control group. Data are displayed as mean ± SD, *n* = 3. One-way ANOVA followed by Tukey's multiple comparisons test was used in each statistical analysis.
