# Supplementary material for: Saikosaponin D Inhibits the Proliferation and Promotes the Apoptosis of Rat Hepatic Stellate Cells by Inducing Autophagosome Formation
Source: Evid Based Complement Alternat Med. 2021 Aug 18;2021:5451758. doi: 10.1155/2021/5451758 (PMC8390134; doi:10.1155/2021/5451758)
Supplement: Supplementary Materials — Figure S1: effects of SSd on nonactivated HSC-T6 cell proliferation. (A) Nonactivated HSC-T6 cell proliferation was detected under SSd treatment for 48 h using EdU kit and flow cytometry. (B) Percentage of proliferating cells based on EdU flow cytometry detection. The ns means no significant difference versus the control group. Data are displayed as mean ± SD, n = 3. One-way ANOVA followed by Tukey's multiple comparisons test was used in each statistical analysis. Figure S2: effects of SSd on nonactivated HSC-T6 cell apoptosis. (A) Nonactivated HSC-T6 cell apoptosis was detected under SSd treatment for 48 h using Annexin V-FITC PI double-stained kit and flow cytometry. (B) Percentage of apoptotic cells based on Annexin V-FITC PI double-stained flow cytometry detection. The ns means no significant difference versus control group. Data are displayed as mean ± SD, n = 3. One-way ANOVA followed by Tukey's multiple comparisons test was used in each statistical analysis. [file 5451758.f1.docx]

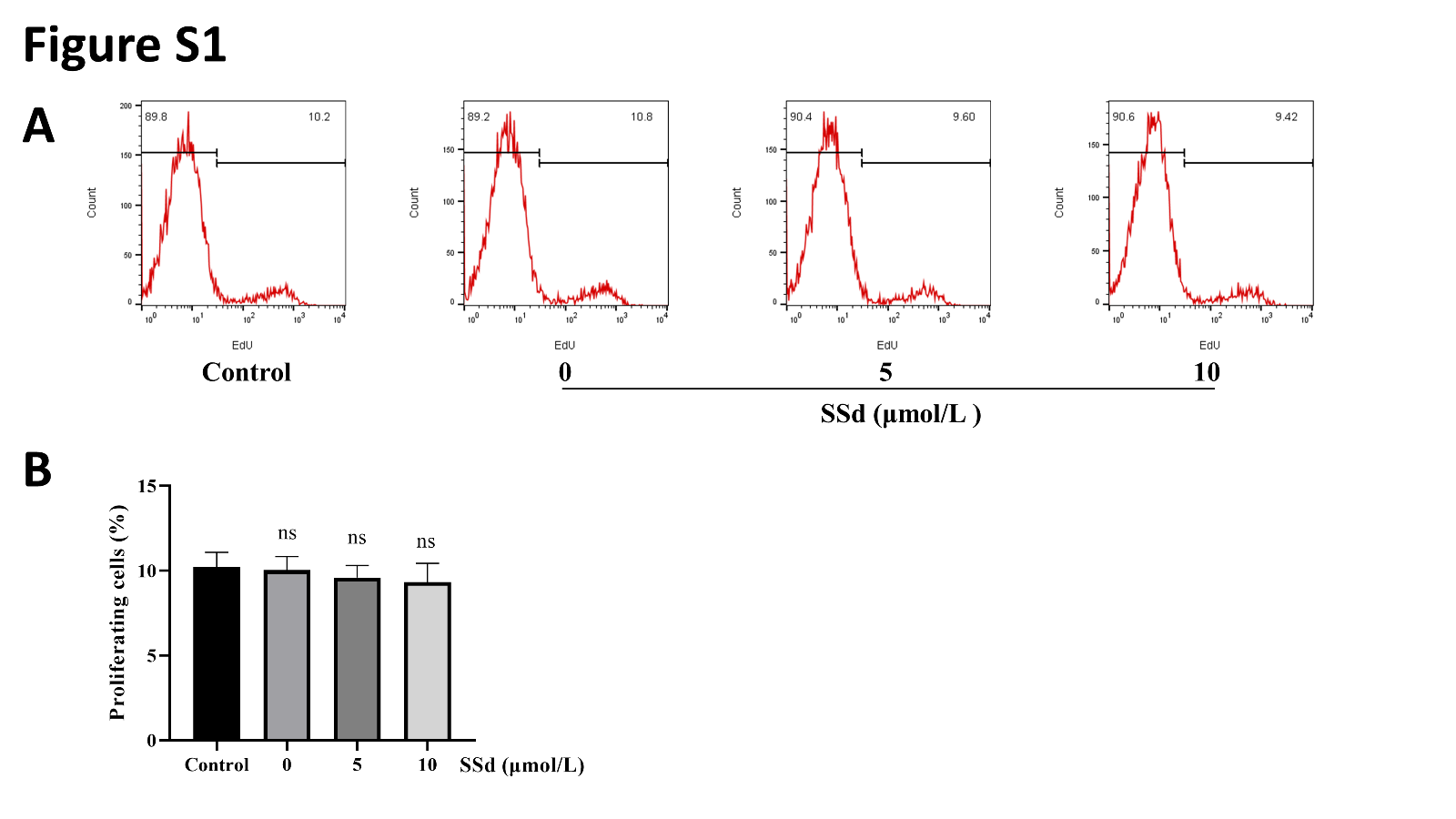


Figure S1. Effects of SSd on non-activated HSC-T6 cell proliferation.

(A) Non-activated HSC-T6 cell proliferation was detected under SSd treatment for 48 h using EdU kit and flow cytometry. (B) Percentage of proliferating cells based on EdU flow cytometry detection. The **ns** means no significant difference versus the control group. Data were displayed as mean ± SD, n = 3. One-way ANOVA followed by Tukey's multiple comparisons test was used in each statistical analysis.


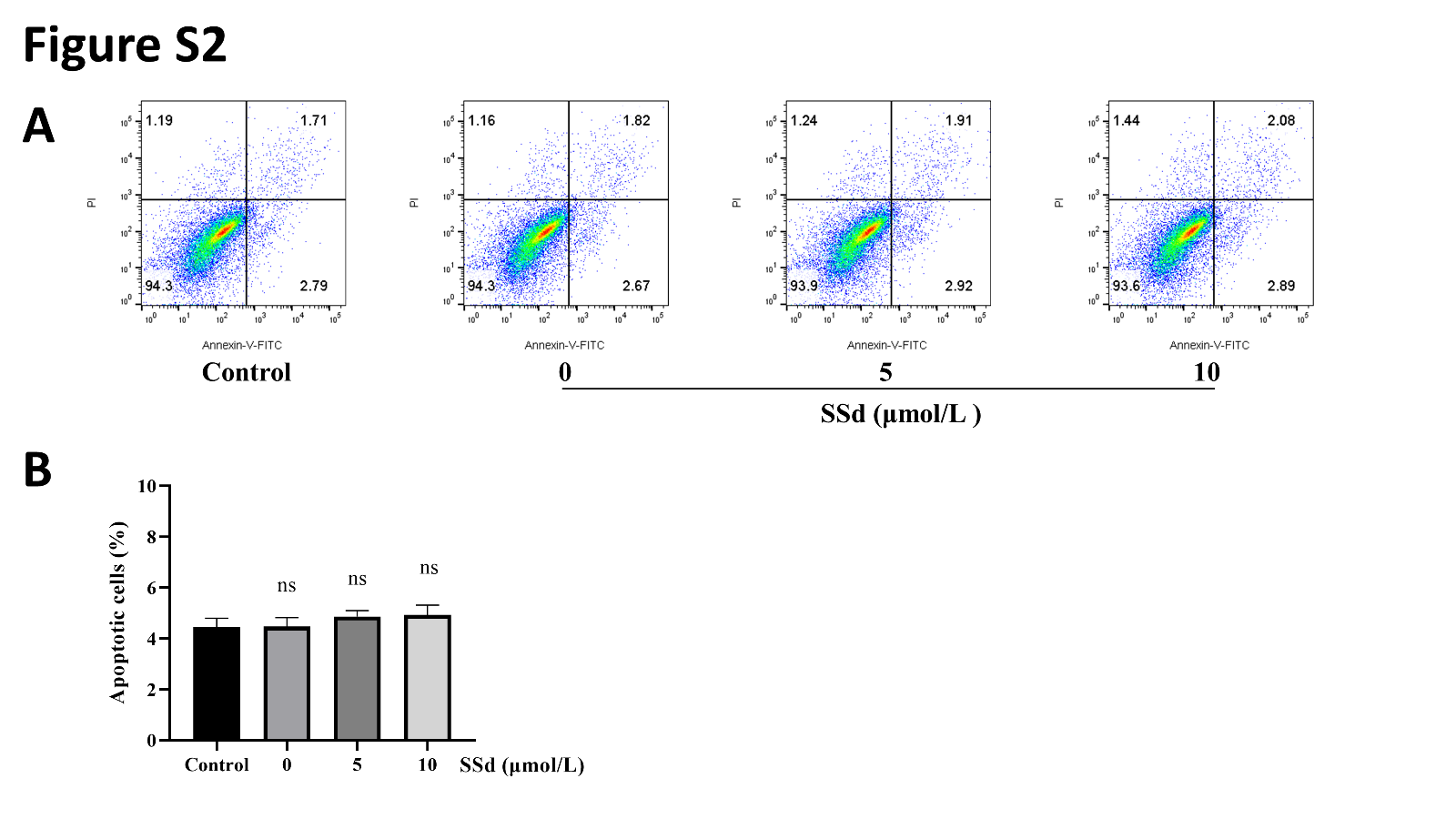


Figure S2. Effects of SSd on non-activated HSC-T6 cell apoptosis.

(A) Non-activated HSC-T6 cell apoptosis was detected under SSd treatment for 48 h using Annexin V-FITC PI double-stained kit and flow cytometry. (B) Percentage of apoptotic cells based on Annexin V-FITC PI double-stained flow cytometry detection. The **ns** means no significant difference versus control group. Data were displayed as mean ± SD, n = 3. One-way ANOVA followed by Tukey's multiple comparisons test was used in each statistical analysis.
